# Assessment of 10-nm Particle Number (PN) Portable Emissions Measurement Systems (PEMS) for Future Regulations

**DOI:** 10.3390/ijerph17113878

**Published:** 2020-05-30

**Authors:** Barouch Giechaskiel, Tero Lähde, Sawan Gandi, Stefan Keller, Philipp Kreutziger, Athanasios Mamakos

**Affiliations:** 1 European Commission, Joint Research Centre (JRC), 21027 Ispra (VA), Italy; tero.lahde@ec.europa.eu; 2 Sensors Europe GmbH, 40699 Erkrath, Germany; sawan.gandi@sensors-europe.eu; 3 AIP GmbH Co. KG, 87490 Haldenwang, Germany; stefan.keller@aip-automotive.de; 4 Horiba Europe GmbH, 61440 Oberursel, Germany; philipp.kreutziger@horiba.com; 5 AVL List GmbH, 8020 Graz, Austria; athanasios.mamakos@avl.com

**Keywords:** air pollution, vehicle emissions, real-driving emissions (RDE), portable emission measurement systems (PEMS), particle measurement programme (PMP), sub-23 nm, solid particle number, catalytic stripper, regeneration

## Abstract

The particle number (PN) emissions of vehicles equipped with particulate filters are low. However, there are technologies that can have high PN levels, especially below the currently lower regulated particle size of 23 nm. Sub-23-nm particles are also considered at least as dangerous as the larger ultrafine particles. For this reason, the European Union (EU) is planning to regulate particles down to 10 nm. In this study we compared prototype portable emission measurement systems (PEMS) and reference laboratory systems measuring from 10 nm. The tests included cycles and constant speeds, using vehicles fuelled with diesel, gasoline or liquefied petroleum gas (LPG). The results showed that the PEMS were within ±40% of the reference systems connected to the tailpipe and the dilution tunnel. Based on the positive findings and the detection efficiencies of the prototype instruments, a proposal for the technical specifications for the future regulation was drafted.

## 1. Introduction

The pollution from vehicles can have important health consequences [1]. There is a growing concern in the public health community about the contribution of ultrafine (<0.1 μm) particles to human health due to their higher deposition fraction, deeper penetration, and higher retention rate in the lungs [2,3]. The average daily exposure is approximately 10^4^ p/cm^3^ but can exceed 10^5^ p/cm^3^ in some cities [4,5]. The sources contributing to the particle number emissions can be different to those contributing to the mass emissions [6]. In most cities the major ultrafine particle number source is road traffic [6,7]. In the last 15–20 years, both mass and number showed significant reductions [8]. Estimations also show that they will continue decreasing in Europe until 2030 [6]. Policies (e.g., Euro emission standards), traffic management, and fleet restrictions (e.g., low emission zones) have contributed significantly to these reductions [8]. Particulate matter levels are currently being focused on because there is a strong association between increases in particulate matter concentration and mortality rates due to COVID-19 [9,10].

In the European Union (EU) the particle number (PN) emissions of solid particles >23 nm have been controlled since 2011 for diesel light-duty vehicles and since 2014 for gasoline direct injection vehicles [11]. Heavy-duty vehicles emissions have been controlled since 2013 [12]. The laboratory type approval of vehicles was augmented by on-road real-driving emissions (RDE) testing with portable emissions measurement systems (PEMS) [13]. The studies with PN PEMS have significantly increased over the last years [14,15,16]. PEMS, due to their small size and simpler design, may have higher measurement uncertainty compared to the laboratory grade equipment [17,18]. PEMS measure undiluted exhaust gas with all the associated challenges (e.g., condensation, wear of instruments) [19]. In contrast to studies assessing instruments for ambient air or personal exposure (e.g., [20,21,22,23,24,25]), the assessment of PN PEMS is limited [13,26,27,28,29]. The studies gave maximum differences of 50% (light-duty) to 65% (heavy-duty) to the laboratory systems for the majority of the cases. This measurement uncertainty is taken into account when the vehicles are assessed on the road. For example, a light-duty vehicle in the laboratory must respect the 6 × 10^11^ p/km limit, but for the on the road RDE assessment, a result below 1.5 × 6 × 10^11^ p/km is still acceptable.

The 23-nm lower size of the current regulation methodology misses a large part of emitted particles for some technologies, as reviews on sub-23-nm emissions from vehicles have shown [30,31]. For example, studies have shown that the particles <23 nm could be as many as those >23 nm for gasoline vehicles [11]. Concerns have also been raised for CNG (compressed natural gas)-fuelled vehicles [13,32]. Particles smaller than 23 nm have higher deposition efficiency in the human respiratory system and sometimes have different chemical composition (e.g., higher percentage of metal oxides), thus need also attention [31,33]. Horizon 2020 projects worked on developing sampling systems and instruments for measuring reliably below 23 nm [34,35,36]. In parallel, the informal Particle Measurement Programme (PMP) group of the Working Party on Pollution and Energy (GRPE) developed a protocol that extended the lower size to approximately 10 nm for the laboratory systems [37,38]. The intention is to include the new methodology in the Global Technical Regulation (GTR) 15 for light-duty vehicles and in the near future apply it in the EU regulation. However, the research for PEMS fulfilling the future requirements is limited [39]. Most importantly, it is still not known how much the measurement uncertainty will be affected by the extension of the lower size from 23 nm to 10 nm. 

The objective of this study is to assess experimentally the measurement uncertainty of prototype PEMS measuring from 10 nm. Systems from four instrument manufacturers are compared with laboratory systems measuring particles emitted from modern Euro 6 vehicles. The results of this study provide the basis of the future technical specification of 10-nm PN PEMS.

## 2. Materials and Methods 

The tests were conducted at the one-axle vehicle emissions laboratory (VELA 1) of the Joint Research Centre (JRC) of the European Commission in Italy. An original equipment manufacturer (OEM) bi-fuel port fuel injection (PFI) gasoline–liquefied petroleum gas (LPG) vehicle, a diesel-fuelled vehicle equipped with diesel particulate filter (DPF) and a gasoline direct injection (GDI) vehicle were tested; all Euro 6c, coded as PFI, LPG, DPF and GDI. Market fuels were used for the tests. The diesel fuel (B7) had cetane number 54.3, 7.1 mg/kg sulphur, 3.6% polyaromatics, 6.5% FAME (fatty acid methyl esters) from rapeseed. The gasoline fuel (E10) had RON (research octane number) 95.2, 7.5 mg/kg sulphur, 30% aromatics, and 5.8% ethanol content. The test cycles were the worldwide harmonized light vehicles test cycle (WLTC) with cold or hot engine start, and constant speeds. The DPF vehicle was once actively regenerated via connecting to the ECU (electronic control unit) of the vehicle with a diagnostic maintenance tool. 

**Figure 1:**
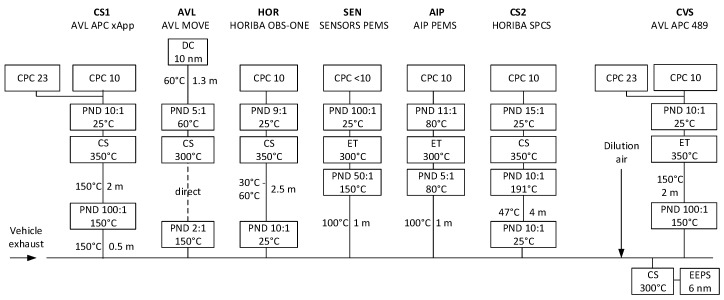
Schematic of the setup. CPC = condensation particle counter; CS = catalytic stripper; DC = diffusion charger; EEPS = engine exhaust particle sizer; ET = evaporation tube; PND = article number diluter.

The experimental setup is plotted in Figure 1. Two reference systems and four PEMS between them were connected to the tailpipe. Another reference system was connected to the dilution tunnel. All systems were designed for the future regulation that will require measurements from approximately 10 nm. In order to put results into perspective and confirm the accuracy of the setup, one of the reference systems at the tailpipe and the reference system at the dilution tunnel were additionally equipped with 23-nm counters. The system at the dilution tunnel with the 23-nm counter was fulfilling the current regulation requirements. A short description of the systems follows; more details are shown in Figure 1.

One of the reference systems at the tailpipe was the APC × App from AVL (Anstalt für Verbrennungskraftmaschinen List) (Graz, Austria). It diluted the sample in a hot diluter (150 ℃), and then thermally pretreated it in a catalytic stripper (CS) [40] at 350 °C. A cold dilution decreased the temperature and brought the concentrations to appropriate levels for the CPCs (from AVL): a model 488-10 with 75% efficiency at 10 nm and a model 488-23 with 56% efficiency at 23 nm. A 50 cm heated line (150 ℃) was used to connect the APC xApp to the tailpipe. This system is abbreviated as CS1.

The second reference PN system at the tailpipe was the SPCS-2110 (Horiba, Kyoto, Japan), which was initially modified in the PEMs4Nano project to allow sub-23-nm measurements [41]. A prediluter heated to 191 °C and using filtered air at approximately ambient temperature was connected directly to the tailpipe. A 4 m heated line (47 °C) brought the diluted aerosol to a hot diluter at 191 °C. A catalytic stripper (CS) at 350 °C and a secondary dilution then followed. The thermally pretreated aerosol was measured with a CPC from TSI (Thermo Systems Inc.) (Shoreview, MN, USA) with 75% detection efficiency at 10 nm. This system is abbreviated as CS2.

The AVL MOVE PEMS was connected directly at the tailpipe diluting the sample with a 2:1 hot dilution at >150 °C [39]. An evaporation tube in series with a catalytic stripper (both set at 300 °C) and a secondary dilution 5:1 at 60 °C followed. The diluted sample was transferred to the particle detector (Automotive Partector, from Naneos, Windisch, Switzerland) with a 1.3 m heated line at 60 °C. The principle of the detector was based on the aerosol measurement with induced currents [42]. A fraction of particles charged in a corona charger was periodically removed by a pulsed electric field. The rate of change of the aerosol space charge was then determined in a Faraday cage by a noncontact measurement. The detector was modified to shift the 50% detection size toward 10 nm by means of modifying the pulsing electric field. This system is abbreviated as AVL and was the only diffusion charger (DC) based system.

The Horiba on-board system (OBS-ONE) PN PEMS was a modified version of the NanoParticle Emission Tester (NPET) (from TSI) optimized to minimize particle losses [43,44]. The first diluter (10:1) was located directly at the sample probe at the tailpipe. A 2.5 m heated line between 30 °C and 60 °C, an optimized for losses heated catalytic stripper at 350 °C, and a second dilution (9:1) followed. The detector was an isopropyl alcohol-based CPC with 50% efficiency approximately at 10 nm. This system is abbreviated as HOR.

The condensation particle number (CPN) PEMS module from Sensors (Saline, MI, USA) consisted of a sampling probe with a heated line at 100 °C, a hot diluter at >150 °C (dilution 50:1), an evaporation tube (300 °C) instead of a catalytic stripper, a second stage diluter 50:1 to 100:1, and a CPC with 92% efficiency at 10 nm from Sensors. This system is abbreviated as SEN.

The AIP (Automotive Industry Products) PEMS consisted of a 1 m heated line at 100 °C, a hot diluter (5:1) at 80 °C, an evaporation tube at 300 °C and a secondary diluter (11:1) at 80 °C. The CPC was an isopropyl alcohol-based TSI model 3007 modified to achieve 75% efficiency at 10 nm. This system is abbreviated as AIP.

The reference PN system at the dilution tunnel with constant volume sampling (CVS) was an APC 489 (AVL) [45], similar to the APC xApp, having an evaporation tube instead of a catalytic stripper. The 23-nm CPC was a 3790 model from TSI while the 10-nm CPC was a model A-20 from Airmodus (Helsinki, Finland). This system is abbreviated as CVS.

An EEPS (engine exhaust particle sizer) spectrometer model 3090 from TSI was measuring the size distributions from 5.6 nm to 560 nm downstream of a catalytic stripper. An additional simple mixing dilution (5:1) was used to provide sufficient flow for the EEPS. The EEPS charged the particles in a unipolar charger, classified them depending on their electrical mobility, and measured their current with 22 electrometers in real time.

## 3. Results

The size distributions of the vehicles and the >23-nm emission results will first be presented, followed by the results of the 10-nm systems. 

### 3.1. Size Distributions and >23-nm Results

**Figure 2:**
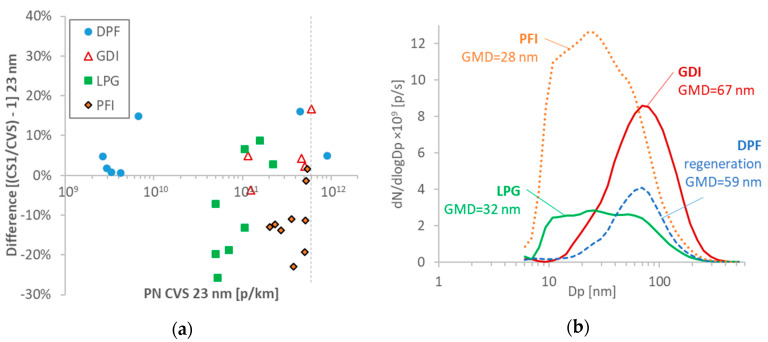
Basic results: (**a**) comparisons of 23-nm particle number (PN) reference systems at the tailpipe and the dilution tunnel (CVS). Each point is a test cycle. The dotted vertical line gives the laboratory PN emissions limit; (**b**) examples of size distributions for the various vehicles, measured with the engine exhaust particle sizer (EEPS) downstream of a catalytic stripper (CS) at 300 °C and multiplied with the CVS flow rate to make the concentrations comparable. DPF = diesel particulate filter; GDI = gasoline direct injection; GMD = geometric mean diameter; LPG = liquefied petroleum gas; PFI = port fuel injection.

The comparison of the 23-nm systems for the various vehicles is plotted in Figure 2a. The differences ranged from −25% to +20%, with no particular trend with the emission levels.

Figure 2b plots examples of size distributions for the various vehicles. The DPF and GDI vehicles emitted big particles (geometric mean diameters (GMDs) 50–70 nm), while the PFI and LPG smaller with GMDs <40 nm. 

### 3.2. Efficiencies

**Figure 3:**
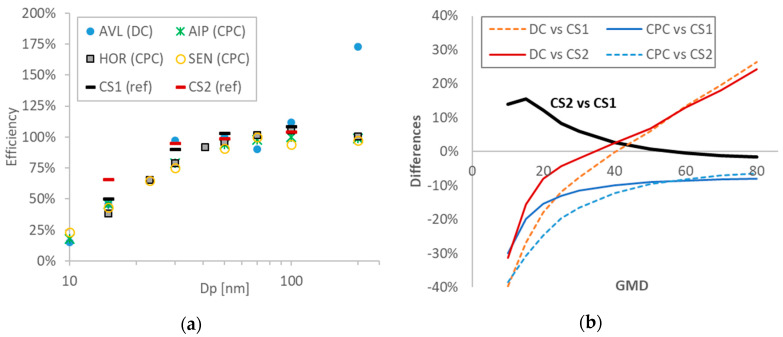
Comparisons of systems: (**a**) Efficiencies of the systems in function of the (monodisperse) mobility diameter; (**b**) calculated differences among the instruments in function of the (polydisperse) geometric mean diameter (GMD). CPC = condensation particle counter; CS = catalytic stripper; DC = diffusion charger.

The efficiencies of all 10-nm systems (i.e., normalized penetration to the plateau) as provided by the instrument manufacturers, are plotted in Figure 3a. The efficiencies were around 100% at diameters between 50 nm and 200 nm. The only exception was the DC-based system (AVL) which had 175% efficiency at 200 nm. At 15 nm, the efficiencies were around 30–50%, with the exception of the reference system CS2 which had 64% efficiency. At 10 nm, all PEMS had <25% efficiency. 

Based on these efficiencies, the expected differences among the instruments were calculated for different geometric mean diameters (GMD) (Figure 3b). Beginning with the two reference systems (CS1 and CS2), the calculated differences were within 5% at GMDs >40 nm and reached 15% at 15–20 nm. The DC-based system (AVL) differences from the reference systems were calculated from −30% to +30%, with an optimized performance at approximately 40 nm. The CPC-based systems (HOR, AIP, SEN) could underestimate around 10% the emissions for GMDs >40 nm, reaching 40% at small GMDs (10 nm).

### 3.3. Reference Systems

**Figure 4:**
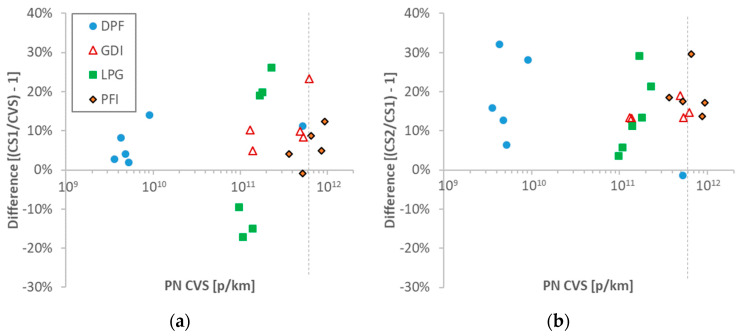
Comparisons of 10-nm particle number (PN) reference systems: (**a**) tailpipe versus dilution tunnel (CVS); (**b**) tailpipe CS1 versus tailpipe CS2. Each point is a test cycle. The dotted vertical lines show the laboratory PN emissions limit (for >23 nm). CS = catalytic stripper; DPF = diesel particulate filter; GDI = gasoline direct injection; LPG = liquefied petroleum gas; PFI = port fuel injection.

The differences of the reference systems of this experimental campaign are plotted in Figure 4. The results are plotted in function of the system at the dilution tunnel (CVS) because this sampling location is expected to remain as the reference point in the future regulation. 

Figure 4a plots the difference of tailpipe CS1 from the dilution tunnel CVS system. The differences were typically within 15%, with some tests reaching 25% for the LPG vehicle. It should be emphasized that the two systems were from the same instrument manufacturer and the penetration curves were relatively close.

Figure 4b compares the two reference systems at the tailpipe. The differences were up to 30%, with a mean difference of 15%. The differences were a little bit higher from the expected theoretical differences (Figure 3b). The GDI and the DPF vehicles with the largest particle sizes (GMDs 50–70 nm), did not show much lower differences compared to the PFI and LPG vehicles (25–35 nm) (13% vs. 18%). For these differences in GMDs, an effect of 10% would be expected between the two CS systems for the different vehicles. Due to the high scatter of the deviations, this effect was not evident. Thermophoretic or agglomeration losses in the 0.5 m heated tube to the CS1 inlet were estimated to be typically 1–2%, and always <5%, suggesting that they were also not the reason for the differences. Thus, the absolute level of the calibration of the instruments was the main reason for the differences and not their size-dependent losses.

### 3.4. PEMS

**Figure 5:**
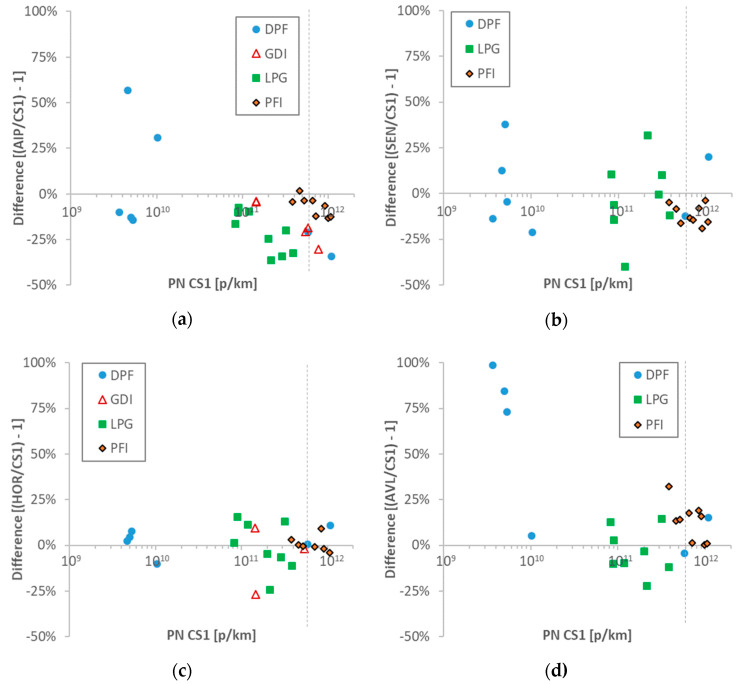
Comparisons of 10-nm particle number (PN) portable emissions measurement systems (PEMS) to reference CS1: (**a**) AIP; (**b**) Sensors (SEN); (**c**) Horiba (HOR); (**d**) AVL. Each point is a test cycle. The dotted vertical lines show the laboratory PN emissions limit (for >23 nm). CS = catalytic stripper; DPF = diesel particulate filter; GDI = gasoline direct injection; LPG = liquefied petroleum gas; PFI = port fuel injection.

Figure 5 compares the PEMS with the reference system at the tailpipe (CS1). Comparisons with the remaining reference systems will be presented later. The results are plotted separately for each car and test cycle. 

The AIP was measuring lower between −40% and 0% with a few exceptions at low emission levels <10^10^ p/km for the DPF equipped vehicle (Figure 5a). One of the main reasons was that the system reached its maximum range during cold start spikes, as will be discussed later. The SEN was measuring within ±35% (Figure 5b). The SEN GDI results were excluded because the instrument inlet flow rate was low due to the underpressure at the tailpipe caused by the suction of the dilution tunnel (−10 mbar). Most tests with the HOR were within ±15%, with only two tests at −25% (Figure 5c). The AVL was measuring within ±25% with the exception of the tests at low emission levels with the DPF-equipped vehicle (Figure 5d). Note that the results with the GDI vehicle were excluded due to sampling error of the system.

Interestingly, there was no clear size dependency of the results for all PEMS. This had to do with the similar efficiencies of the PEMS with the reference system CS1. Furthermore, there was no trend with the concentration levels. The differences were similar for the whole range examined, from 2 × 10^12^ p/km to the detection limit of the instruments. Close to their lower detection limits, the AIP (Figure 5a) and SEN (Figure 5b) had higher scatter (around 50%), and the DC-based system (AVL) 100% (Figure 5c). Note that the SEN was used with the high dilution setting (50 × 100) for all tests. The HOR remained within 25% down to 5 × 10^9^ p/km (Figure 5d). 

### 3.5. Real Time Signals

Some examples of typical instruments responses are presented in this section.

**Figure 6:**
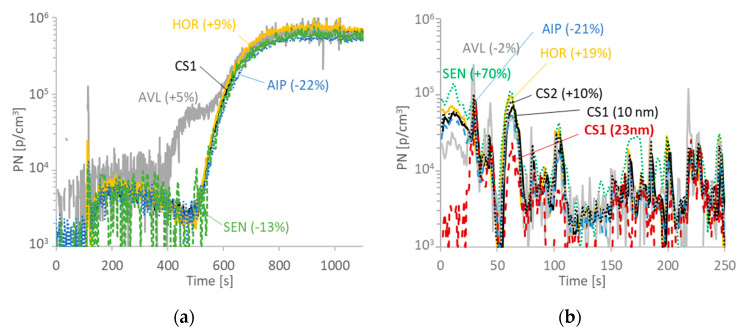
Particle number (PN) concentration examples for the DPF vehicle: (**a**) regeneration; (**b**) cold start.

Figure 6a presents the active regeneration of the DPF. The maximum temperature at the tailpipe reached 280 °C. The two reference systems (CS1 and CS2) were within 2%. The 23-nm concentration was only 23% lower than the 10-nm counter of CS1, indicating that the majority of the particles were large (see also Figure 2b). The PEMS were very close as well (max deviation, AIP −22%). Of interest is the deviation of the AVL system at 400–600 s, where the exhaust gas temperature was around 60–150 °C. This will be further discussed in the “Discussion” section. The contribution of this overestimation was 17% to the total regeneration emissions.

Figure 6b presents the cold start part of a WLTC. It should be emphasized that the absolute levels, even for the first 250 s, were very low, 30 times below the PN limit (1.9 × 10^10^ p/km). As it has been shown elsewhere, during the first idling seconds there were mainly particles below 23 nm [44,46]. This is clearly evident by the huge difference between the CS1 10-nm and CS1 23-nm signals. Nevertheless, PEMS were quite close to the CS1 system. The PEMS deviations could be attributed to differences in the penetration curves at small particles and the thermal preconditioning of the sample by the PEMS. The differences in these first 250 s were within 20%, with the exception of the SEN system that was overestimating +70% (partly due to the high dilution used). The AVL system showed on average a 2% difference over the 250 s, although, it showed larger deviations in individual parts (measuring lower concentrations at idling and reporting higher concentrations during some accelerations). 

**Figure 7:**
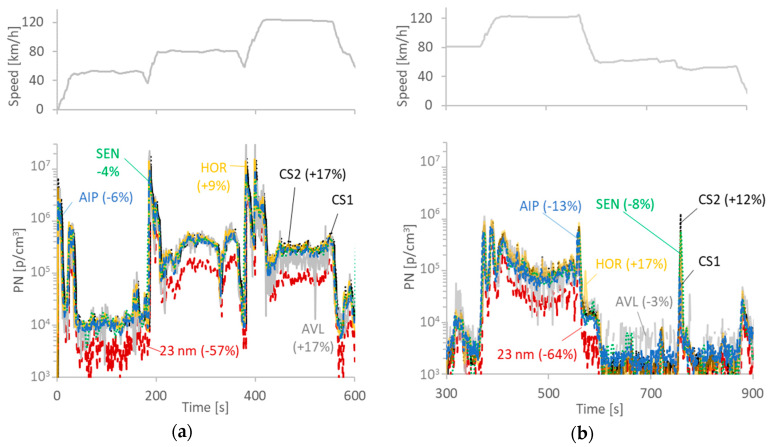
Particle number (PN) concentration examples for: (**a**) PFI vehicle; (**b**) LPG vehicle. Upper panels plot the speed profiles.

Figure 7a plots the results for the PFI vehicle during constant speed modes with relatively hard accelerations between the speeds. The high concentrations during the accelerations are evident. The agreement of all instruments was very good (within 17%), even though there was a significant amount of particles below 23 nm. The AVL instrument was noisier, and in some cases, slightly lower than the rest instruments (see at 120 km/h, around time 500 s). However, the mean difference from the reference system was similar to the other PEMS.

Figure 7b plots the results for the LPG fuel during constant speed modes. The emissions were low with the exception of the high-speed mode (120 km/h) and during engine breaking (e.g., 550 s and 750 s). These particles could be mainly oil particles as has been discussed in the literature [47,48]. The agreement of the instruments was also good for this example (within 17%).

**Figure 8:**
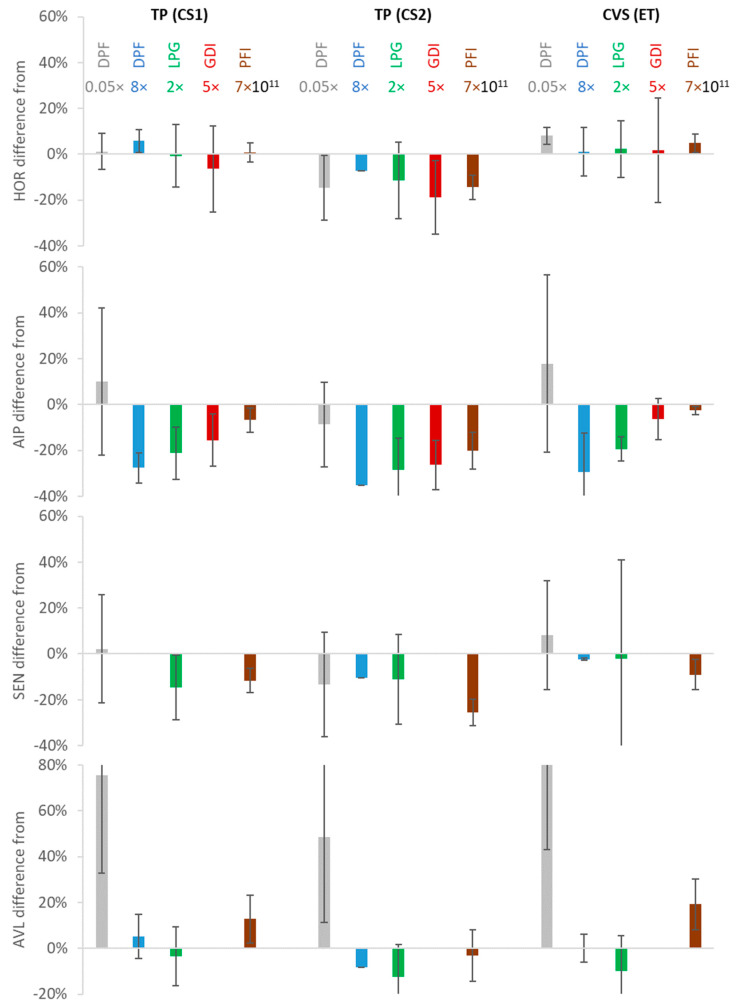
Differences of PEMS from the three reference systems at the tailpipe (TP) and the dilution tunnel (CVS). Error bars show one standard deviation. CS = catalytic stripper; DPF = diesel particulate filter; ET = evaporation tube; GDI = gasoline direct injection; LPG = liquefied petroleum gas; PFI = port fuel injection.

Figure 8 summarizes the differences of the PEMS compared to the three reference systems. For each PEMS and reference instrument combination, the results are separated to the different vehicles. The DPF vehicle is further separated to low and high emissions. An indication of the average emissions for each vehicle is also given. The best performing PEMS (HOR) was within ±25% from all reference systems, while the rest were within ±20–40%. Higher differences were seen only close to the detection limit of the systems, at two orders of magnitude below the PN limit (DPF vehicle, <10^10^ p/km). Depending on the actual lower detection limit of the instruments, the difference at <10^10^ p/km ranged from 25% to 50% for CPC-based systems and 50% to 100% for the DC-based system. 

## 4. Discussion

In this study we compared prototype 10-nm PEMS with prototype 10-nm laboratory systems. Even though the systems are commercially available, there are no technical requirements for such systems, because they are not regulated yet. The PMP group is working on finalising the specifications for the laboratory systems and the specifications of the on-road systems (PEMS) will depend on those. A lot of work was also done in the Horizon projects dealing with sub-23-nm particle measurements [35,36,49]. Thus, the two reference systems of this study should be representative of the future laboratory systems. An important difference is that the sampling location of the reference systems will be the dilution tunnel and not the tailpipe. For this reason, we had another reference system at the dilution tunnel as well. The dilution tunnel system had an evaporation tube instead of a catalytic stripper as thermal pre-treatment unit. The comparison of the reference systems at the tailpipe and the dilution tunnel gave an indication of the effect of the “sampling location”, i.e., particle losses and exhaust flow influence, which is independent from the accuracy of the PEMS. The comparison of the PEMS with the reference systems at the tailpipe gave an indication of the PEMS accuracy.

The location effect, i.e., the differences between the reference systems at the tailpipe and the reference system at the dilution tunnel, was on average 5–11% for the different cars, with some cases reaching 25%. Such differences are in line with previous findings and are due to particle losses from the vehicle tailpipe to the dilution tunnel and uncertainties on the determination of the exhaust flow [50,51]. The comparison of the two reference systems at the tailpipe gave an average difference of 15%, with differences reaching 30%. Regarding PEMS accuracy, the best performing PEMS (HOR) was within ±25% from all reference systems, while the rest PEMS were within ±20–40%. The results are very promising and confirm that 10-nm PEMS methodology is ready for introduction in the regulation. It is worth mentioning that the differences between PEMS and reference systems were at the same levels that the reference systems had between them. 

The differences of PEMS versus reference systems in this study did not show any trends with the geometric mean diameters (GMDs) of the emitted particles. For example, vehicles with small (LPG, PFI) or large (GDI, DPF) GMDs, or events with high or low sub-23-nm fraction (e.g., engine breaking or cold start) had similar differences. This was partly expected, because for CPC-based systems, the expected size effect would be <10%, based on the efficiencies of the systems and the GMDs (25 to 70 nm) of the vehicles of this study. For the DC-based system, a 20–30% effect would be expected, but it was not the case. For the 30 min tests, the mean differences were similar for all vehicles.

Even though the GMDs in this study were from 25 to 70 nm, their standard deviation was large, but still in line with the reported in relevant reviews (1.9–2.0) [11,52]. Cases with separate narrow solid particles mode peaking below 25 nm could result in higher differences between the instruments. In this case, the monodisperse efficiencies are a good estimation of the expected differences (for 15 nm, the PEMS efficiencies ranged from 38% to 46%, very near to that of the reference system—50%). Thus, the differences would be still within experimental uncertainties. Higher differences would be seen in comparison to the second reference (66% efficiency at 15 nm). An example of the effect of the efficiencies with two laboratory systems measuring the exhaust of a moped was discussed in another study [53]. The efficiencies at 15 nm were 50% and 85% and their results had >50% differences when the GMD was <25 nm. Even though the 85% efficiency is extreme, such systems will be allowed in the future 10-nm regulation; Horizon 2020 projects have shown that such efficiencies are possible [36]. Cases with GMDs smaller than 25 nm, have been reported for mopeds and motorcycles [54], but also for a few gasoline vehicles [11,55] and CNG (compressed natural gas)-fuelled vehicles [12]. For diesel vehicles, the mean size is typically large [56,57], with the exception of cold start [46]. As other studies have discussed, the quality of the fuel and the bio content can also have an impact on the emissions due to the different volatility [58]. This could have an effect on the mean size but in particular on the cold start. Similarly, the PEMS should be evaluated at different ambient temperatures in the future. Finally, for heavy-duty engines, the influence of crankcase ventilation emissions should also be assessed. Studies addressing these topics so far have focused on the laboratory systems [32] or the 23-nm PEMS [28].

It should be noted that the evaluation was done with the dilution tunnel connected at the tailpipe of the vehicles. This created a small underpressure (<10 mbar) at the inlet of the instruments, which is the opposite of what the instruments are typically exposed to (slight overpressure during on-road tests). This setup though is what is recommended in the regulation for the validation of the PEMS, i.e., their comparison with the laboratory equipment. The maximum underpressure permitted is 12.5 mbar, thus our tests should be representative of future validations, with the exception that many instruments were used in series. For the SEN instrument, this underpressure resulted in a very low inlet flow rate which gave warnings with the GDI vehicle. Yet, for the SEN instrument, the GDI results (which were excluded) were ±45% (±35%) from the reference instrument (CS1). This underpressure might have negatively affected the accuracy of the remaining instruments (e.g., at determination of the dilution ratio). Nevertheless, there was no such indication of reduced accuracy for the remaining PEMS in our tests.

The agreement of PEMS and reference systems was excellent also between the real time signals, at least for the CPC-based systems. The real time signal of the DC-based system (AVL) had slightly bigger deviations especially at spikes. It is likely that its high response time (<1 s for DC- vs. 3.5 s of the other systems) contributed to this behaviour. The DC real time signals would be more comparable with an internal averaging of approximately 3 s. The size dependency of the DC-based system did not reveal any trends. For example, the vehicles with small GMDs (PFI and LPG) had similar differences with the DPF vehicle, which emitted larger particles. Only at the beginning of the regeneration a small shoulder appeared that was not evident at the other CPC-based instruments, or at the dilution tunnel. Such a shoulder has not appeared at other regenerations with this instrument at previous campaigns [39]. Differences of the sample thermal preconditioning can be excluded because the AVL had a catalytic stripper at 300 °C, similar to some of the other PEMS. Large particles could only partly explain such deviation. For example, even if all particles were 200-nm particles, the overestimation would be 100% (see Figure 3a), and not >1000% (see Figure 6a). Furthermore, the size distribution measurements at the dilution tunnel did not show an increase of the mean diameter, thus it is unlikely that this overestimation was from larger particles. Such deviations at such low emission levels have been reported at heavy-duty vehicles during urea injection [59]. They were explained by the relatively high mean positive charge of the particles formed inside the SCR (selective catalytic reduction of NO_x_) at elevated temperatures [60]. The AVL positive unipolar charger could not properly condition their positive charge, leading to overestimation of their concentration. The contribution of these particles decreased as the neutral soot emissions increased during and after the regeneration, improving the overall correlation. The urea injection and the temperature at the SCR is not known at our study, so this assumption would need further studies to be confirmed. In any case the contribution toward the final result was only 17%. A technical solution to address such charged particles, which is based on a combination of negative and positive corona, was successfully evaluated for heavy-duty applications [59]. Nevertheless, the mean deviations from the reference systems were at the same levels as with the rest CPC-based systems, indicating that calibration uncertainties are still more important. Even though the PEMS efficiency curves seem to be very close to each other, the calibrations were done at different laboratories with different calibration instruments. The calibration instruments have an uncertainty of 5%, which could translate to a 10–15% uncertainty of the PEMS calibration (efficiency, but also dilution ratios) [61]. The same applies for the reference laboratory systems that were used in this study, but for PEMS the uncertainty could be higher because the procedures are not standardized. This uncertainty is representative of the uncertainty in the field because the calibrations are typically done at the instrument manufacturers and not at the users’ laboratories.

The different thermal preconditionings of the PEMS (temperatures 300 °C or 350 °C, evaporation tube or catalytic stripper) did not reveal any particular deviations from the reference instruments. Cold dilution with catalytic stripper (HOR), to hot dilution with an evaporation tube (AIP) had equivalent behaviour and any differences were mainly due to calibration uncertainties. Nevertheless, the necessity (or lack thereof) of a catalytic stripper needs further studies, especially considering that the laboratory systems will be required to have a catalytic stripper in the future 10-nm regulation.

This study confirmed the challenges that tailpipe sampling has: (i) high PN concentrations, (ii) exhaust flow determination and (iii) condensation spikes [39,51]. All these risks existed also with the 23-nm PEMS and are not solely related to the 10-nm PEMS. Some of the risks may need more attention with the 10-nm protocol. For example, the peak concentrations can reach 1 × 10^8^ p/cm^3^, which is double the concentration that was necessary for the 23-nm PEMS. One of the systems (AIP) was saturated at some of the cold starts, where the emissions had spikes of 5–8 × 10^7^ p/cm^3^. The maximum concentration of the system was 2 × 10^7^ p/cm^3^, but it will be increased to 1 × 10^8^ p/cm^3^ in the future. The need for higher maximum concentrations for 10-nm measurements was also recently identified during the evaluation of another PN PEMS [39].

The condensation spikes might also be more critical with the lower size. However, at our results they affected equally the 23-nm and 10-nm counters when they appeared at the reference CS1 system during a steady state test with the GDI vehicle. The contribution was 1.5 × 10^11^ particles, which is equivalent to 1% of the PN limit. The spikes were accompanied with a big sample pressure drop that could trigger an instrument warning alarm in the future. Condensation spikes did not appear at the PEMS or the dilution tunnel instruments for this test, indicating that they do not necessarily originate from big droplets from the vehicle for example, but are setup- and instrument-specific. Another case was the first test with the DPF vehicle with the AIP system. The water trap was full in the previous LPG tests and there was a warning. At some point during the test, some spikes appeared, but their concentration level was very low (1.7 × 10^11^ particles, i.e., 1% of the PN limit). One more case was with the SEN system during the first GDI test, but these results were excluded due to the low inlet flow rate of the instrument. No other condensation spikes appeared at other PEMS or tests. A previous study showed that the condensation spikes can contribute up to 5% at the PN limit of 6 × 10^11^ p/km [39]. However, at lower ambient temperatures anticipated during RDE testing, they may appear more often and may have higher magnitude. Cold spots should be avoided to eliminate or minimize this issue.

Based on the previous findings, Table 1 summarizes the efficiencies of the systems and the proposed limits for the future regulation. The (upper) requirement at 200 nm was left at 200% as in the past, because the contribution of the 200-nm particles to the range of interest (around 30–70 nm) is negligible. Most studies have shown that the modern vehicles emit particles with GMDs in the 30–70-nm range [11,39]. The values in the mid-range became much more stringent (±15%) than the current tolerances for 23-nm systems (±30% tolerances in the mid-range). It is expected that the smaller tolerances can counterbalance the additional uncertainty that smaller particle sizes introduce due to the wider range of efficiencies of the systems. The requirements for the cut-off curve range (15 nm) was set to 30–70% in order to include the laboratory references, otherwise they could be further narrowed.

## 5. Conclusions

We compared four prototype portable emissions measurement systems (PEMS) with reference laboratory systems that measure solid particle number (PN) emissions from 10 nm. The best performing PEMS was within ±25% of the reference systems connected both at the tailpipe and the dilution tunnel, and the rest of the PEMS were within ±40%. The differences were at similar levels that the reference had: on average ±15%, with some tests reaching 30%. The PEMS limit of detection was found to be below 10^10^ p/km, i.e., 50 times below the laboratory PN limit 6 × 10^11^ p/km. At these low levels, the condensation particle counter (CPC) systems had 25% to 50% differences from the reference systems, while the diffusion charger system had 50% to 100% differences. There was no apparent indication of size dependent differences, even though the geometric mean diameters of the vehicles tested spanned from 25 nm to 70 nm, indicating that calibration, flow and dilution control are still important contributors to the uncertainty. This measurement campaign confirmed that PEMS are ready to be introduced in the future regulations and the future efficiency requirements were drafted. Next steps include transferring the remaining technical specifications from the 23-nm regulation (e.g., linearity, volatile removal efficiency), and addressing topics such as verification against charged particles, alarms for condensates, and instrument deterioration.

## Figures and Tables

**Table 1:** Experimental and proposed efficiencies.

Diameter	10 nm	15 nm	30 nm	50 nm	70 nm	100 nm	200 nm
Ref experiments	-	0.50–0.66	0.90–0.95	1.00–1.03	-	1.04–1.08	-
PEMS experiments	0.15–0.23	0.38–0.46	0.75–0.97	0.91–1.00	0.90–1.02	0.94–1.12	0.97–1.73
PEMS proposed ^1^	0.10–0.50	0.30–0.70	0.75–1.05	0.85–1.15	0.85–1.15	0.80–1.20	0.80–2.00

^1^ Some margin for experimental calibration uncertainties and production variability.
